# Follicular helper T-cell lymphomas: disease spectrum, relationship with clonal hematopoiesis, and mimics. A report of the 2022 EA4HP/SH lymphoma workshop

**DOI:** 10.1007/s00428-023-03607-5

**Published:** 2023-07-27

**Authors:** Sarah L Ondrejka, Catalina Amador, Fina Climent, Siok-Bian Ng, Lorinda Soma, Alberto Zamo, Stefan Dirnhofer, Leticia Quintanilla-Martinez, Andrew Wotherspoon, Lorenzo Leoncini, Laurence de Leval

**Affiliations:** 1 Pathology and Laboratory Medicine Institute, Cleveland Clinic, Cleveland, OH USA; 2 Division of Hematopathology, Department of Pathology and Laboratory Medicine, University of Miami, Miami, FL USA; 3 Pathology Department, Hospital Universitari de Bellvitge, IDIBELL, L’Hospitalet De Llobregat, Barcelona, Spain; 4 Department of Pathology, Yong Loo Lin School of Medicine, National University of Singapore, Singapore, Singapore; 5 Department of Pathology, City of Hope National Medical Center, Duarte, CA USA; 6 Institute of Pathology, University of Würzburg, Würzburg, Germany; 7 Institute of Medical Genetics and Pathology, University Hospital Basel, University of Basel, Basel, Switzerland; 8 Institute of Pathology and Neuropathology, Eberhard Karls University of Tübingen and Comprehensive Cancer Center, University Hospital Tübingen, Tübingen, Germany; 9 Department of Histopathology, The Royal Marsden Hospital, London, UK; 10 Department of Medical Biotechnology, University of Siena, Siena, Italy; 11 Institute of Pathology, Department of Laboratory Medicine and Pathology, Lausanne University Hospital and Lausanne University, Lausanne, Switzerland

**Keywords:** T-cell lymphoma, TFH lymphoma, Angioimmunoblastic T-cell lymphoma, Peripheral T-cell lymphoma, Clonal hematopoiesis, High-throughput sequencing, EA4HP workshop

## Abstract

Follicular helper T-cell lymphomas (TFH lymphomas) were discussed in session V of the lymphoma workshop of the European Association for Haematopathology (EA4HP)/Society for Hematopathology (SH) 2022 meeting in Florence, Italy. The session focused on the morphologic spectrum of TFH lymphoma, including its three subtypes: angioimmunoblastic-type (AITL), follicular-type, and not otherwise specified (NOS). The submitted cases encompassed classic examples of TFH lymphoma and unusual cases such as those with early or indolent presentations, associated B-cell proliferations, or Hodgkin/Reed–Sternberg-like cells. The relationship between TFH lymphoma and clonal hematopoiesis was highlighted by several cases documenting divergent evolution of myeloid neoplasm and AITL from shared clonal mutations. The distinction between TFH lymphoma and peripheral T-cell lymphoma, not otherwise specified (PTCL, NOS), was stressed, and many challenging examples were presented. Various cases highlighted the difficulties of differentiating TFH lymphoma from other established types of lymphoma and reactive conditions. Cutaneous T-cell lymphoma expressing TFH markers, particularly when resulting in lymph node involvement, should be distinguished from TFH lymphomas. Additional immunophenotyping and next-generation sequencing studies were performed on various cases in this session, highlighting the importance of these technologies to our current understanding and classification of TFH lymphomas.

**Supplementary Information:**

The online version contains supplementary material available at 10.1007/s00428-023-03607-5.

## Introduction

T follicular helper (TFH) cells represent a functional subset of CD4-positive lymphocytes that are specialized providers of B-cell help. In normal immune responses, TFH cells are necessary for the formation and maintenance of germinal centers (GCs) and interact with GC B cells, aiding in their differentiation towards antibody-producing plasma cells and memory cells [1]. In 2007, immunophenotyping and gene expression profiling analyses identified the TFH cell as the cell of origin (COO) of angioimmunoblastic T-cell lymphoma (AITL) [2–4], and this specific cellular derivation has become a cardinal defining feature of the disease [5] Over time, other peripheral T-cell lymphomas (PTCLs) have been recognized to similarly express immunophenotypic markers (CD10, BCL6, CXCL13, PD1, and ICOS) and/or gene expression signatures characteristic of TFH cells [2–4, 6–10]. In the 2017 revised 4^th^ WHO classification, an umbrella category of “nodal lymphomas of TFH origin” was created to include AITL, which remains the prototypic TFH lymphoma, and two related entities sharing TFH features, namely, follicular TCL and PTCL with TFH immunophenotype [11]. The concept of related entities unified by common clinical, immunophenotypic features has been reinforced by genetic findings [8, 12–14] and carried over to new classification schemes. The ICC-2022 recognizes follicular helper T-cell lymphoma as a single entity comprising three subtypes, angioimmunoblastic-type (AITL), follicular-type, and not otherwise specified (NOS) [15]. The proposed WHO5-2022 classification is slightly different, considering a family of three distinct nodal T-follicular helper cell lymphomas with similar suffixes, i.e., angioimmunoblastic-type, follicular-type, and NOS [16].

The central theme of the 2022 EA4HP/SH workshop in Florence was “Provisional and Emerging Disease Entities.” Forty-two cases were submitted for session V, dedicated to the topic “Lymphomas with TFH Phenotype.” Specifically, the call requested cases illustrating the borderlands of TFH lymphoma with PTCL, NOS and reactive conditions, and the association with clonal hematopoiesis (CH). The final diagnoses fell into three categories. Twenty-five cases represented examples of TFHL, 11 cases were concluded as other PTCL entities or lymphoproliferative disorders with TFH antigen expression, and six cases consisted of reactive or other lymphoproliferations.

## Follicular helper T-cell lymphomas

The 25 cases ultimately classified as TFH lymphoma (TFHL) comprised 16 cases of AITL, 3 follicular-type (TFHL-F), and 6 NOS (TFHL-NOS) (Supplemental Table 1). The 25 patients with TFHL were 13 males and 12 females, with a median age of 68 years (range 30 to 80 years). Among 13 patients with detailed information on the presenting symptoms, interestingly, only one presented with a complete constellation of general symptoms and biological abnormalities classically reported in association with AITL. Seven patients presented with clinically silent disease, and the other five patients had various non-specific complaints [17, 18].

Except for one tonsillectomy, all submitted biopsies were from lymph nodes (LN). These comprised 19 surgical excisions and 5 core biopsies, reflecting increasing reliance on fast and minimally invasive small-volume biopsies for lymphoma diagnoses [19–21]. The submitted core biopsies were sufficient for diagnostic testing in all cases, including high-throughput sequencing (HTS) analyses performed in three cases. However, four patients with a final diagnosis of TFHL made on excision had one or several needle core biopsies preceding the contributive excisional biopsy. In two of these patients, the cores had led to an erroneous diagnosis (of Hodgkin lymphoma in one case and EBV-positive B-cell lymphoproliferative disorder in another case), and in two patients, the core biopsies were non-conclusive and likely delayed the diagnosis. Only one case with a core biopsy was submitted with unstained slides to the workshop, emphasizing how a lack or paucity of archived material from core biopsies precludes the possibility of tissue-based research and compromises potential future clinical needs [22].

**Fig. 1:**
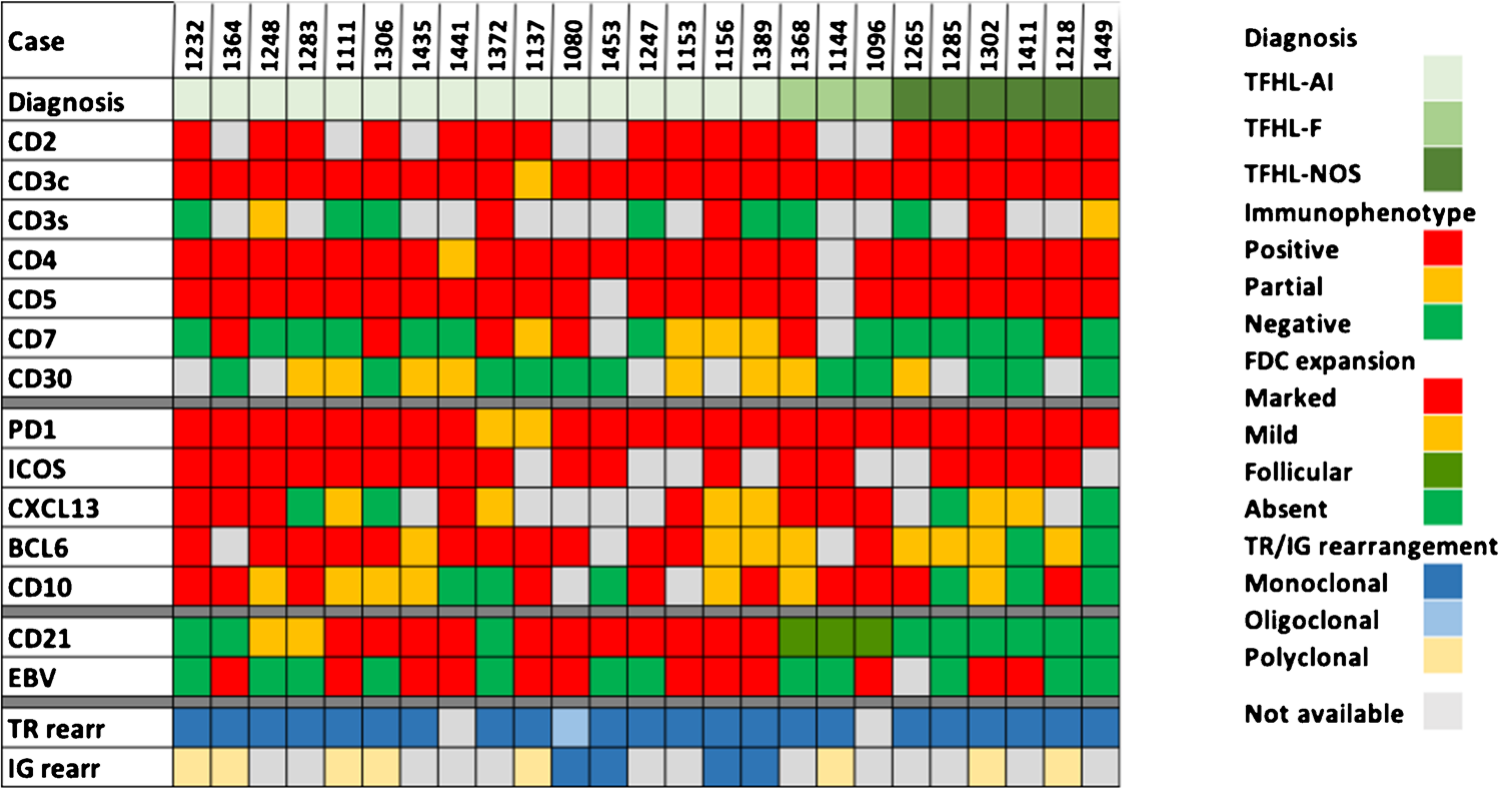
Heatmap representation of the immunophenotype, EBV, and clonality results in the 25 follicular helper T-cell lymphomas (TFHLs) of session V. By immunohistochemistry, the neoplastic T cells were positive for CD2 (18/18), cCD3 (25/25, dim in one case), CD4 (24/24, dim in one case), and CD5 (23/23). The aberrant CD3-/dim CD4+ population reported as a common feature of TFH lymphoma [43] was confirmed in this cohort, as 8/12 cases analyzed by flow cytometry were negative or dim for sCD3 expression. Dim, partial, or negative CD7 expression was found in 17/23 cases. Most cases (17/25), including virtually all of those analyzed by flow cytometry (11/12) showed an aberrant T-cell phenotype regarding pan T-cell antigen expression. Some degree of CD30 positivity in the neoplastic cells was reported in 8/19 cases. Considering the five TFH markers currently recommended as a systematic IHC panel [15, 16], PD1 and ICOS were tested in most cases—25 and 18 cases, respectively—and were usually strongly expressed. The expression of CXCL13, BCL6, and CD10 was more heterogeneous. CXCL13 was interpreted as negative in 4/18 cases, partially positive in 6, and strongly positive in 8 cases. BCL6 expression was found in 20/22 cases but was often partial or weak. CD10 was the least sensitive TFH marker, positive in 17/23 cases, but only weak or partial in 7 cases. Only 50% of the cases in this series, including 9/16 AITL, contained EBV-positive B-blasts. A monoclonal T-cell receptor gene (TR gene) rearrangement was demonstrated in 22/23 cases tested, and the remaining case had an oligoclonal TR gene rearrangement. Four of 11 cases tested for an IG gene rearrangement had a B-cell clone

**Fig. 2:**
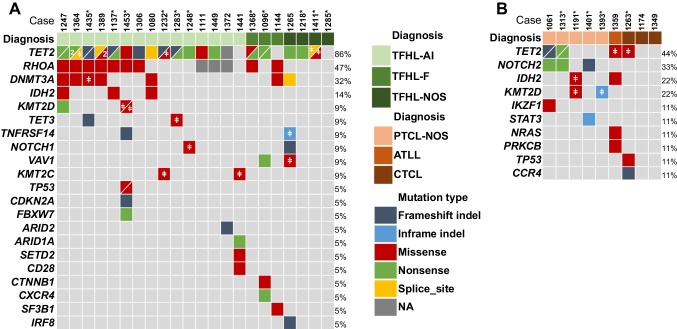
Summary of mutated genes in **A** 22 patients with follicular helper T-cell lymphoma and **B** 8 patients with other T-cell lymphoma types. Genes mutated in at least two patients and/or with one probably pathogenic (ACGS class 4) or pathogenic (ACGS class 5) mutation are shown. For each gene, the percentage of patients carrying mutation(s) is indicated to the right of the table. High-throughput sequencing results were provided by the submitters, or generated by the panel (*, see supplementary information). ǂ denotes ACGS class 3 mutations (variants of unknown significance). Of note, the assays used for genetic profiling did not assess potential gene fusions, for example, those involving *CD28*, *ICOS*, and *VAV1,* which represent other oncopathogenic mechanisms in TFHL [13, 91]

Immunophenotypic features, EBV status, and clonality results of the 25 TFHL cases are summarized in Fig. 1. HTS analysis was performed in 22 cases either by the submitters (12 cases) or by the panel (10 cases) (Fig. 2A, Supplementary information and Supplementary Table 2). Several studies have highlighted the characteristic mutational landscape of TFH lymphomas, which typically associates mutations in epigenetic regulators (*TET2*, *DNMT3A*, and *IDH2)*, *RHOA* G17V, and alterations in the T-cell receptor (TCR) signaling pathway [12, 23–26]. In this small cohort of workshop cases, *TET2* was the most frequently mutated gene in 21 out of 24 cases, often with 2 or 3 mutations. *RHOA* mutation (G17V hotspot) was detected in 9 out of 22 cases, *DNMT3A* in 7 out of 25 cases, and only 3 out of 18 AITL harbored an *IDH2* mutation involving the R172 residue.

The submitted TFHL cases were separated according to their most interesting feature(s) into thematic groups, which addressed the following: the clinical and pathological variety of AITL, the features of TFHL-F and TFHL-NOS, the spectrum of associated B-cell proliferations, and the association of TFH lymphomas with CH and myeloid neoplasms.

### The clinicopathologic spectrum of AITL: partial nodal involvement, early phase, and asymptomatic disease

AITL remains the best-characterized subtype of TFH lymphoma, with distinctive clinical and pathological features [27]. AITL mainly affects elderly adults, with a median age of 65 years. Most patients present with generalized lymphadenopathy (> 80%) systemic symptoms (> 50%), and frequent cutaneous manifestations or involvement of other extranodal sites [28]. Laboratory abnormalities including anemia (often hemolytic and Coombs-positive), polyclonal hypergammaglobulinemia (30%), hypereosinophilia, cytopenia, and various autoantibodies are commonly observed [28–30]. Patients are usually treated with anthracycline-containing combination chemotherapy, and the outcome of the disease is dismal with overall survival rates in the range of 30–35% at 5 years [30]. Morphologically, AITL is composed of atypical medium-sized lymphocytes with often clear cytoplasm, increased vascularity, and a prominent polymorphous microenvironment with eosinophils, plasma cells, histiocytes, and large immunoblastic cells which may be Reed–Sternberg-like [28, 31] with expanded follicular dendritic cell (FDC) meshworks [31, 32]. Three histological patterns are described: pattern-I, with hyperplastic follicles; pattern-II, with regressive follicles; and pattern-III, diffuse, and which is the most commonly encountered [33, 34]. The neoplastic cells are usually CD4+ and show TFH marker expression [2, 33–36].

**Fig. 3:**
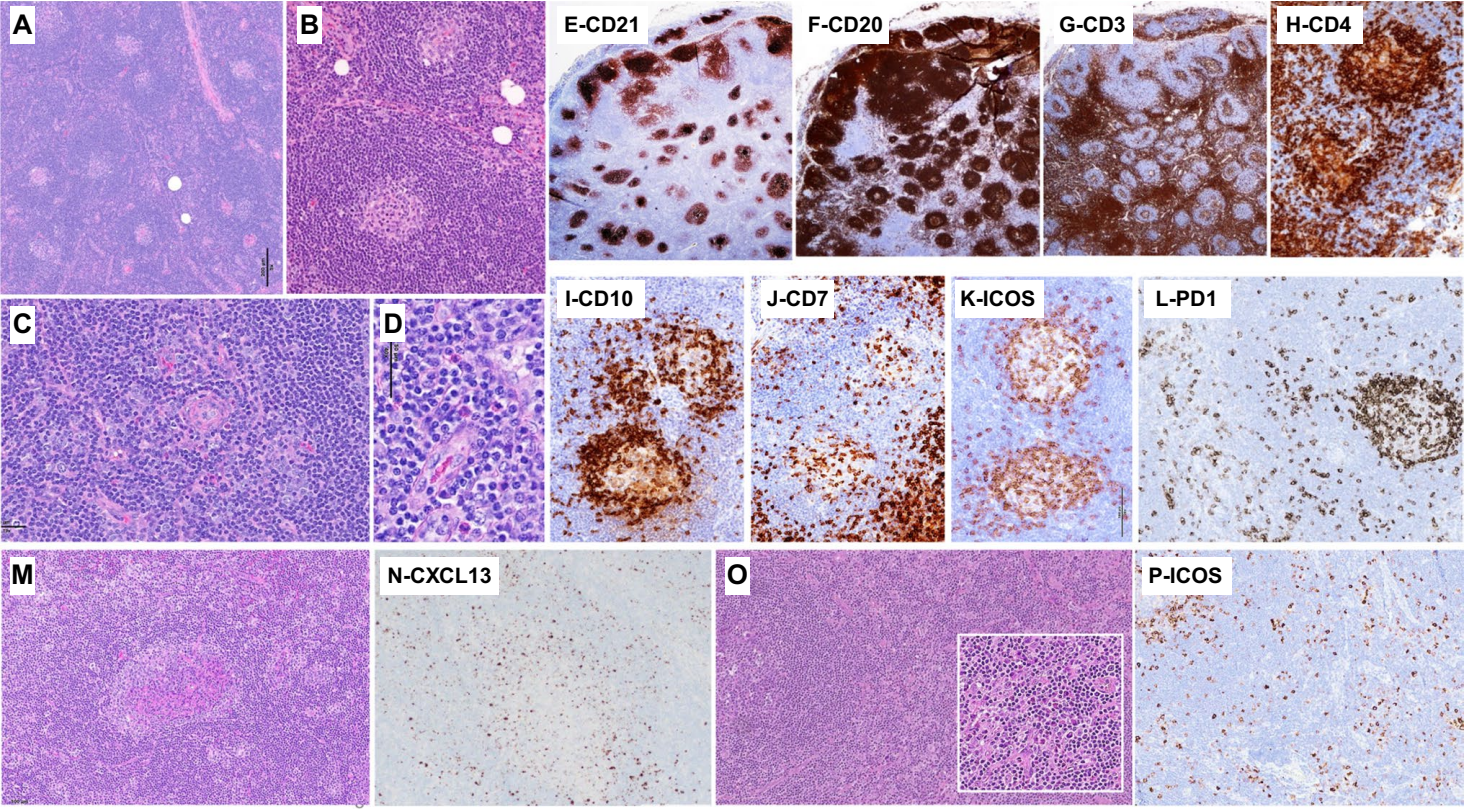
AITL with partial/subtle lymph node (LN) involvement. LYWS-1362 (Pedro Farinha): at low-power view (**A**), the LN presents a globally preserved architecture, with numerous follicles separated by interfollicular areas; (**B**) the follicles have small or regressive GCs and thick mantle zones; (**C**) on close inspection of the interfollicular areas, there is focal increased vascularity and aggregates of slightly atypical cells, also shown in (**D**); panoramic views of the immunostains (**E**–**G**) show CD21+ follicular dendritic cell meshworks confined to follicles (**E**), numerous CD20+ follicles and few extrafollicular B-cells (**F**), while CD3+ cells constitute the majority of extrafollicular cells but also are increased at the periphery of the GCs (**G**); the numerous T cells present at the periphery of the GCs and the inner zone of the mantle zone (**H**–**L**) are positive for CD4 (**H**) strongly CD10+ (**I**), negative for CD7 (**J**), positive for ICOS (**K**), and positive for PD-1 (**L**); note that PD-1 also highlights clusters of extrafollicular cells. LYWS-1248 (Danielle Maracaja): this LN that had a globally preserved architecture contains aggregates of atypical clear cells, distributed at the periphery of the GCs and outside follicles (**M**), and these cells are highlighted by CXCL13 (**N**). LYWS-1283 (Mats Ehinger): this medium magnification of the HE slide shows a follicle (left) with preserved mantle zone, and mildly expanded interfollicular area, containing increased vessels and an atypical cellular infiltrate (inset) (**O**); ICOS highlights cells distributed at the periphery of GCs (upper left) as well as in extrafollicular areas (**P**)

Three cases submitted to the workshop illustrated disseminated neoplasms with limited tumor burden in asymptomatic or pauci-symptomatic patients. Case LYWS-1232, presented by Pedro Farinha (Fig. 3A–L) was most remarkable since after a two-year follow-up without treatment, the patient remained asymptomatic. This 68-year-old woman presented first with localized inguinal adenopathy and a skin rash which resolved after the LN biopsy. Six months later, enlarging LN in several areas prompted another biopsy. Both surgically excised LN showed a preserved architecture comprised of numerous lymphoid follicles with often small, regressively transformed GCs surrounded by uniform but variably expanded mantle zones and fewer hyperplastic GCs. Immunostaining highlighted clusters of CD3+ CD4+ T cells strongly positive for PD1, ICOS, and CD10, and uniformly negative for CD7, confined to the outer edge of the follicles. Flow cytometry on the first biopsy found 3% of the total T cells with an aberrant sCD3- CD4+ CD7- immunophenotype. CD21 and CD23 showed preserved FDC meshworks confined to the follicles, and EBV was not detected. An identical T-cell clone was found in the two biopsies and HTS performed on the second specimen showed only one pathogenic mutation in *TET2*. This case elicited ample discussion regarding how to best diagnose this condition characterized by persistent and disseminated clonal TFH cells not identifiable by morphology alone, in an otherwise hyperplastic LN. Although interfollicular areas were not significantly expanded, with no conspicuous increased vessels, discrete clusters of extrafollicular slightly atypical cells were highlighted by ICOS and PD1. Therefore, the panel favored a diagnosis of AITL, best ascribed to pattern-I and pattern-II; although in this case, the observed features did not completely fit the original description of the different patterns. Indeed, instead of attenuated mantle zones usually reported in the pattern-I, the follicles in case LYWS-1232 had thick mantle zones, with the neoplastic cells distributed between the GCs and the inner aspect of the mantles. Cases LYWS-1248 submitted by Danielle Maracaja (Fig. 3M and N) and LYSW-1383 submitted by Mats Ehinger (Fig. 3O and P) were two other examples of AITL with low neoplastic cell burden and preserved nodal architecture showing the role of immunohistochemistry to highlight atypical TFH cells at the periphery of the GCs and in the interfollicular areas. These two cases also showed preserved, thick mantle zones in the involved follicles. On the contrary, case LYWS-1364 presented by Joan Somja, was more typical of AITL pattern-I. While also characterized by a subtle atypical infiltrate of clear TFH cells around hyperplastic GCs with attenuated mantle zones, the disease occurred in an elderly lady who had polyarthralgia, fever, hypergammaglobulinemia, diffuse lymphadenopathy, slight splenomegaly, and peripheral blood involvement detected by flow cytometry (0.1% circulating CD4+ CD10+ cells). The LN harbored monoclonal TR gene rearrangements, and by HTS mutations in *TET2*, *DNMT3A*, and *RHOA* G17V, the latter with only 1% variant allele frequency, confirming the low neoplastic cell content.

**Fig. 4:**
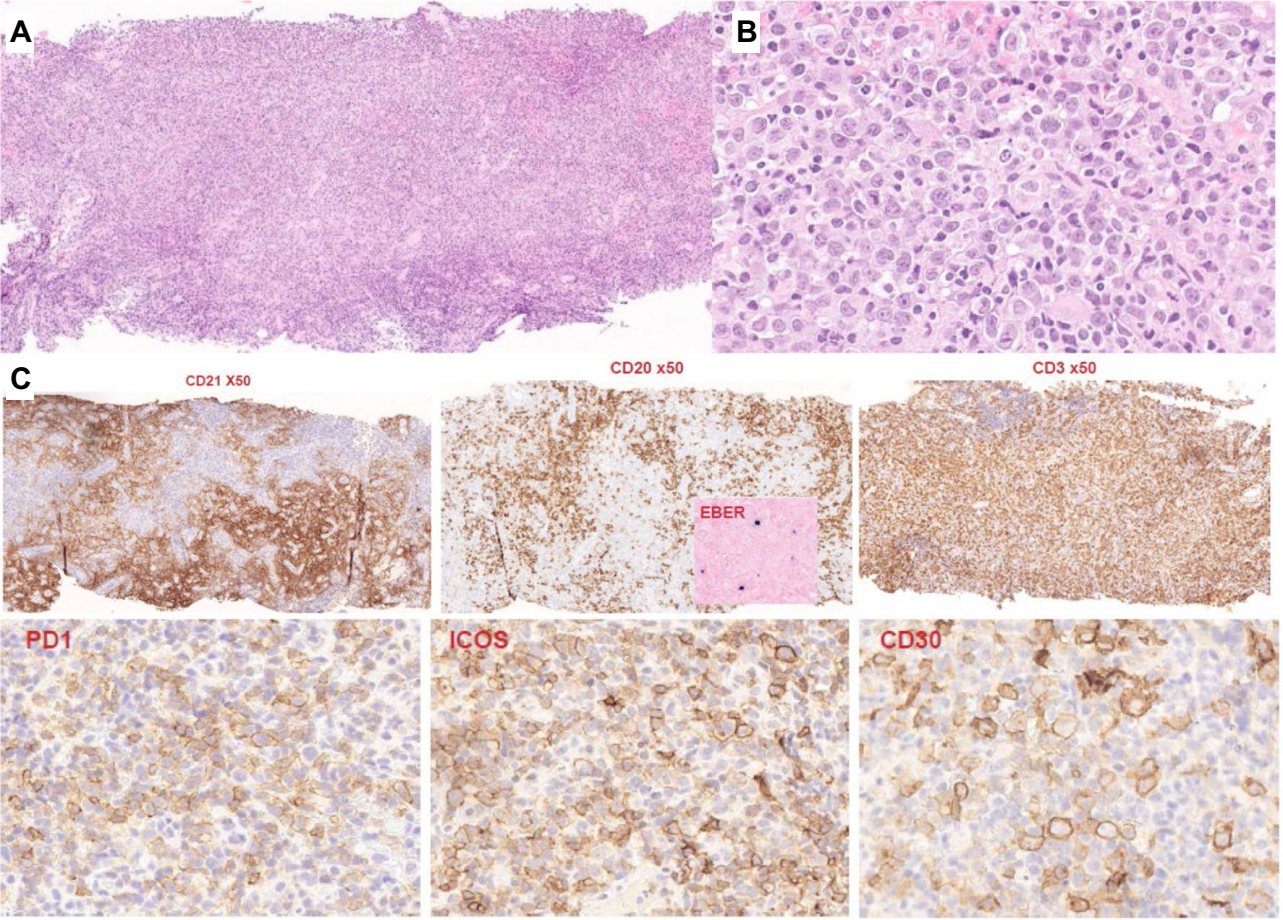
AITL diagnosed on a core needle biopsy. LYWS-1441 (Pierre Isnard): wide LN core needle biopsy, showing an effaced architecture, increased vascularity (**A**) and an atypical cellular infiltrate of clear cells, admixed with small lymphocytes and histiocytes (**B**); on immunostains (**C**), CD21 highlights an expanded meshwork of follicular dendritic cells distributed around increased vessels, CD20 stains residual aggregates of small B-cells, as well as scattered larger B-cells, which are positive for EBV by in situ hybridization (inset), the majority of the infiltrate consists of CD3+ cells, which show extensive expression of PD-1 and ICOS, and CD30 stains the large blastic cells as well as a proportion of the atypical T-cells

Other cases exemplified peculiar clinical features or context. Case LYWS-1435 submitted by Giorgio Croci was another example of indolent AITL. In that case, a first core biopsy had been diagnosed as an atypical lymphoproliferation and re-biopsy after four years without treatment showed histologically typical AITL with mutations in *TET2*, *DNMT3A*, and *RHOA*. In that case, it was not a question of limited tumor burden in the first biopsy, but rather poorly preserved morphology of what was clearly a lymphoma from the beginning, which remained clinically silent for several years. Case LYWS-1366 submitted by Birgitta Sander showed typical AITL histology, but the patient had no generalized symptoms and had reduced immunoglobulin levels. Case LYWS-1441 submitted by Pierre Isnard was an AITL diagnosed in a LN core biopsy in a 66-year-old man eleven years after a kidney transplant. Post-transplant lymphoproliferative disorders are rarely of T-cell type, and most commonly consist of PTCL, NOS or hepatosplenic T-cell lymphoma [37]. Despite the limited sampling, the typical features of AITL were present, including the expansion of FDCs and EBV-positive B-cell blasts. Pathogenic mutations in *CD28*, *ARID1A*, and *SETD2* were identified (Fig. 4).

### TFH lymphoma, follicular-type

The designation of this rare subtype of TFHL refers to a pattern of growth related to follicular structures, lacking the extrafollicular proliferation of FDC and proliferative high endothelial venules characteristic of AITL [9, 38, 39]. It was introduced in the 2008 WHO classification as a subtype of PTCL, NOS (follicular T-cell lymphoma), and was moved in 2016 to the category of nodal PTCL with TFH phenotype [5, 11]. TFHL-F encompasses two morphologic presentations: either a truly follicular pattern, mimicking follicular lymphoma (FL-like), or more commonly a pattern resembling progressive transformation of GCs (PTGC-like). In FL-like TFHL-F, the neoplastic cells feature variable cytomorphology, sometimes with clear cytoplasm, and form well-defined intrafollicular aggregates sustained by a meshwork of FDC. In PTGC-like TFHL-F, aggregates of medium-sized pale or clear atypical neoplastic T cells are distributed within expanded mantle zones in large nodules mainly composed of small IgD+ B cells. Besides the t(5;9)(q33;q22) *ITK::SYK* fusion rather specific to TFHL-F but present in only a minority of the cases [40], the mutational pattern of TFHL-F overlaps with that of AITL [8, 41].

**Fig. 5:**
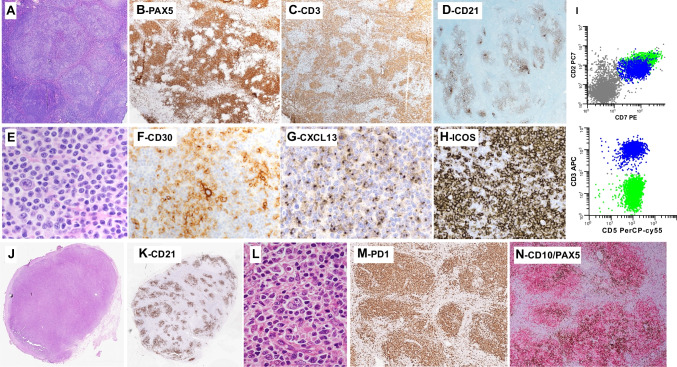
Follicular helper T-cell lymphoma, follicular-type. LYWS-1368 (Emily Mason): in this case, resembling progressive transformation of the GCs, low-power view of the LN shows large nodules, comprising pale cellular aggregates (**A**); PAX5 shows high B-cell content in the nodules, with a moth-eaten appearance (**B**), and CD3 stains the intermixed aggregates of clear cells intermixed within the nodules (**C**); CD21 highlights follicular dendritic cells meshworks underlining the nodules (**D**); on high-power view (**E**–**H**), the pale aggregates comprise atypical medium-sized cells with abundant cytoplasm, and scattered blastic cells (**E**), CD30 stains strongly the large blastic cells (which are also CD20+, not shown) and moderately the atypical clear cells (**F**); the latter shows positive expression of CXCL13 (**G**) and ICOS (**H**); flow cytometry (**I**) demonstrates a population of T cells with an aberrant immunophenotype (sCD3-, CD5+, and CD7+; green dots). LYWS-1096 (Stefano Lazzi): in this follicular lymphoma-like case, a low panoramic view of the LN (**J**) shows a vaguely nodular pattern, which is confirmed by CD21 immunostaining (**K**); the nodules comprised a polymorphic infiltrate including small to medium size atypical cells, large scattered blasts, sometimes resembling Reed–Sternberg cells, and eosinophils (**L**); PD-1 stains the majority of cells in a nodular pattern (**M**); on double immunostaining with CD10 (red) and PAX-5 (brown), the nodules comprised PAX5- CD10+ cells (**N**)

Three cases of TFHL-F (LYWS-1096, 1144, and 1368) were submitted, two with a PTGC-like pattern, and one with FL-like features. The three cases comprised CD21/CD23+ FDC meshworks associated with follicles and CD4+ neoplastic cells that showed a strong TFH immunophenotype (Fig. 1). The three cases also contained CD30+ EBV-positive or CD30+ EBV-negative HRS-like B-cells. This has been reported in the literature as a source of potential diagnostic pitfall with classic Hodgkin lymphoma (cHL) or nodular lymphocyte predominant Hodgkin/B-cell lymphoma [9, 42–44]. Coexpression of CD30 by the neoplastic cells reported in 75% of one case series [44] was conspicuous in only one case (LYWS-1368). The PTCG-like case (Fig. 5A–G) presented by Emily Mason was diagnosed in a patient who had received BCG immunotherapy for bladder cancer and presented with inguinal lymphadenopathy. By staging, the disease was localized. HTS analysis showed two mutations in *TET2* and *RHOA* G17V. The patient was treated by standard polychemotherapy and is alive with no evidence of disease after three years of follow-up. Data in the literature suggest that compared to AITL, TFHL-F more commonly presents as localized stage I/II disease [9, 45, 46]. Whether these might represent a subset of cases with a better prognosis requires further studies. The other two cases of TFHL-F (LYWS-1144 and LYWS-1096 submitted by A. Gray and Stefano Lazzi) presented as disseminated disease. Case LYWS-1096 (Fig. 5H–L) was a good example of the FL-like pattern; it comprised many tumor cells, positive for CD4 and strongly positive for TFH markers and negative for CD7. This case harbored pathogenic mutations in *TET2* (2 mutations), *CXCR4, CTTNB1*, and *VAV1*, but no *RHOA* alteration.

### TFH lymphoma, NOS

TFHL-NOS is the least well-defined subtype of TFHL. It encompasses CD4+ PTCLs expressing a TFH immunophenotype, defined by the expression of at least 2 and ideally 3 TFH markers out of a panel of at least five markers (CD10, BCL6, PD1, ICOS, and CXCL13) that are now recommended to be tested routinely and systematically, any time TFHL might enter into diagnostic consideration [15, 16]. TFHL-NOS may present some features overlapping with those characteristic of AITL, like the presence of EBV+/-B-cell blasts or some increased vascularity, but lacks the typical prominent inflammatory background, vascular proliferation, or FDC expansion of AITL [9, 38, 39].

**Fig. 6:**
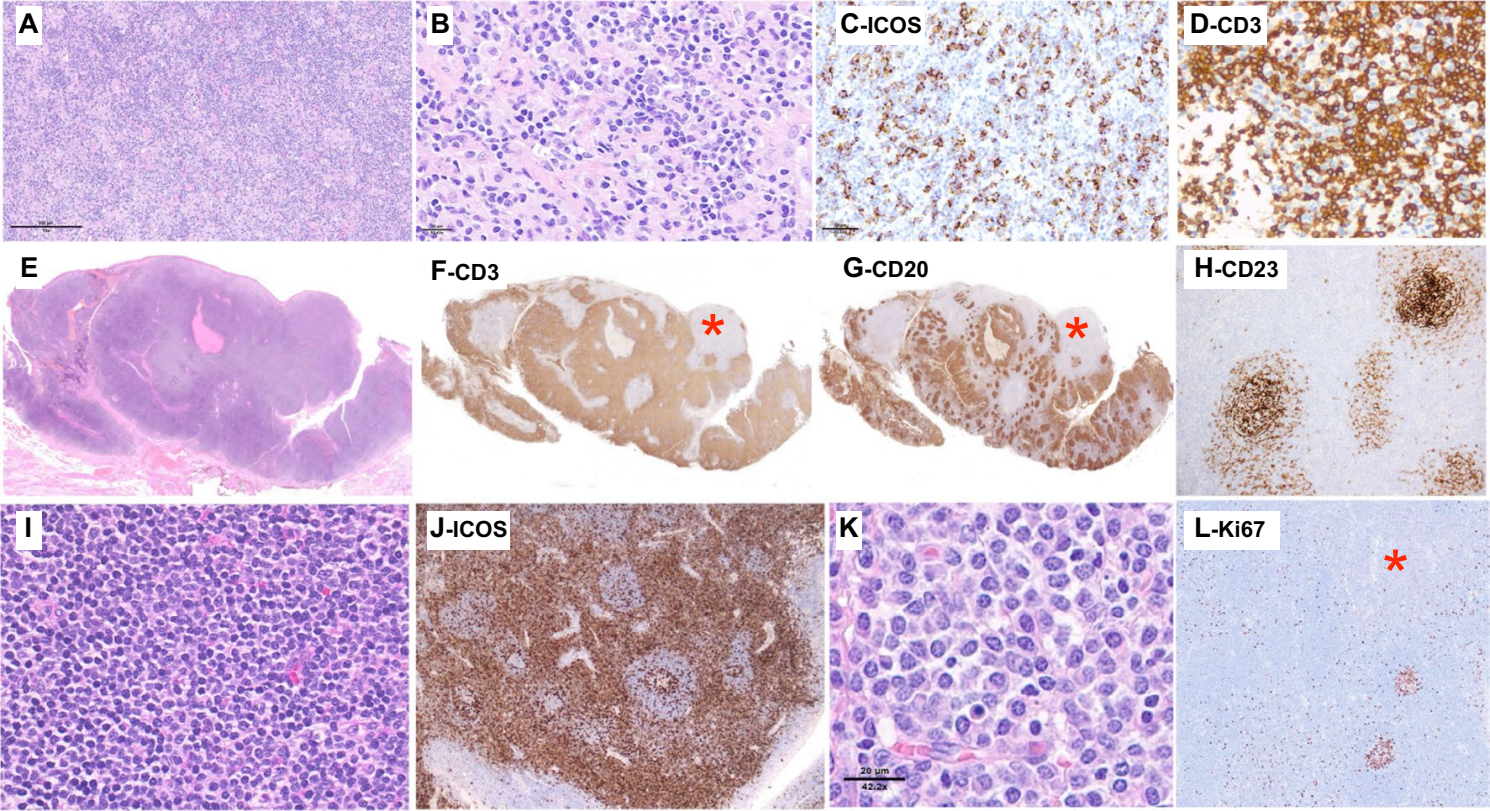
Follicular helper T-cell lymphoma, NOS. LYWS-1302 (Wendy Lim Wen-Hsuan): this LN comprises an epithelioid cell-rich infiltrate (**A**), including atypical lymphoid cells, sometimes with clear cytoplasm (**B**); the atypical cells are positive for ICOS (**C**); and the majority were CD3+ (**D**). LYWS-1218 (Marco Pizzi) in this tonsillectomy specimen, panoramic views (**E**–**G**) show expanded lymphoid tissue, including a marked expansion of CD3+ areas (**F**) and preserved CD20+ follicles (**G**); note the presence of a focus (*) negative for both CD3 and CD20; CD23 immunostaining (**H**) showed follicular dendritic cells restricted to follicles; the T-cell zones (**I**, **J**) comprised a monotonous infiltrate of small to medium-sized lymphoid cells, showing slight atypia being strongly positive for several TFH markers including ICOS shown here (**J**); the area negative for CD20 and CD3 (*) corresponded to sheets of mature plasma-cells (**K**); Ki67 shows very low proliferation index, both in the T-cell zones and in the plasma cells infiltrate (*)(**L**)

Five cases were classified as TFHL-NOS (LYWS-1218, 1265, 1285, 1302, and 1411), four were diagnosed in LN and one in a tonsillectomy. In all cases, the neoplastic cells were CD4+ and expressed at least 2 and often more than 2 TFH markers (Fig. 1). Case LYWS-1302 (Fig. 6A–D) submitted by WH-W Lin was an example of TFHL-NOS with an abundant epithelioid cell content, lacked proliferation of FDCs and contained rare EBV-positive cells. It has been shown that a significant proportion of cases formerly classified as lymphoepitheloid PTCL, NOS (so-called Lennert lymphoma) represent CD4+ neoplasms characterized by a TFH phenotype and are best classified as TFHL [47]. In that case where HTS analysis failed due to insufficient DNA quality, standard cytogenetic analysis had shown an abnormal karyotype.

Case LYWS-1218 submitted by Marco Pizzi (Fig. 6E–L), occured in a 72-year-old patient who had sore throat and bilateral tonsillar enlargement. The tonsillectomy contained atrophic follicles separated by broad T-zones expanded by a monomorphic proliferation of medium-sized CD3+ CD4+ cells, showing little atypia and a strong TFH immunophenotype with minimal micro-environment. In addition, a sharply demarcated area negative for CD20 and CD3 consisted of mature plasma cells, positive for CD138, MUM-1, and IgG but negative for both light chains and for EBV. There was no FDC expansion, and the Ki67 proliferation index was extremely low in both plasma and T-cell compartments. Molecular studies showed monoclonal TRB and TRG, polyclonal IG (IGH, IGK, and IGL) gene rearrangements, and mutations in *TET2*, *IRF8*, *NOTCH1*, *DNMT3A*, *VAV1*, and *TNFRSF14* (Fig. 2A and Supplemental Table 1). The case was submitted with the diagnosis of “indolent TFH proliferation” because the disease was localized by staging, and the patient is still doing very well after 1 year. Given the constellation of morphologic, phenotypic, and genetic findings, the panel favored a diagnosis of TFHL-NOS, acknowledging that a subset of those cases may follow an indolent clinical course or slow progression. Regarding the associated EBV-negative IgG+ light chain-negative polytypic plasma cell proliferation, in the absence of a systemic plasma cell neoplasm, a diagnosis of associated plasmacytoma was favored, given its sharp demarcation from the TFHL, and the lack of a plasma cell component admixed with the TFHL.

### The spectrum of associated B-cell proliferations in TFH lymphoma

The presence of a large B-blastic component, infected or not with EBV, is a characteristic component of AITL, reflected in the name of the entity. These large B-cells, which morphologically may mimic HRS cells, are also often observed in other subtypes of TFHL and are a source of misdiagnosis as HL [42, 48]. Specifically, all three TFHL-F discussed above contained HRS-like cells. Another case of AITL (LYWS-1111 submitted by Joshua Menke) occurred in a patient who had been treated for “atypical HL” and presented with recurrent adenopathy. A microbiopsy of the recurrent LN was interpreted as recurrent cHL, followed by an excision that provided the definitive diagnosis of AITL containing relatively numerous EBV+ large cells. In retrospect, the feature unusual for cHL on the microbiopsy was the strong expression of B-cell markers (PAX5 and CD20) on the CD30+ HRS-like cells. More pronounced B-cell and/or plasma cell proliferations may be observed, polytypic or monotypic, and containing or not EBV, best characterized in AITL but also reported in TFHL-NOS. Transformations to diffuse large B-cell lymphoma in sequential biopsies are well described [32, 42, 49–51].

**Fig. 7:**
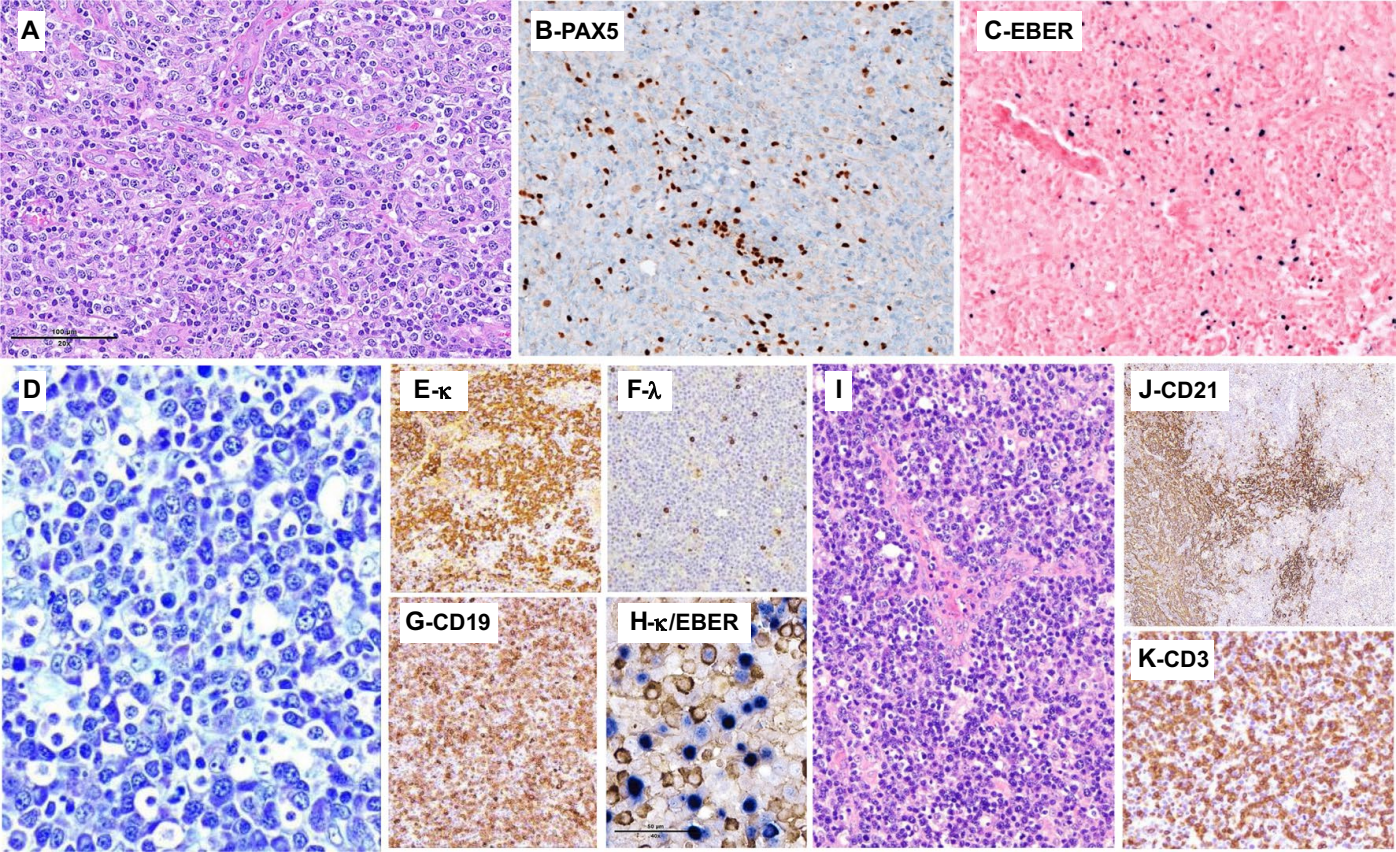
B-cell proliferations in TFH lymphoma. LYWS-1137 (Nives Zimmermann): this TFHL occurring in a particularly young patient (30-year-old) shows a typical morphology of AITL, including prominent arborising blood vessels, and prominent clear cells (note that this case harboured an *IDH2* mutation) (**A**); PAX5 highlighted a moderate number of large nuclei (**B**) that were positive for EBER by in situ hybridization (**C**). LYWS-1080 (Katrin Kurz): this LN comprises a prominent lymphoplasmacytic infiltrate (**D**) containing many plasmacytic cells monotypic for kappa (**E**) and with only very few lambda-positive cells (**F**), as well as numerous B cells highlighted by CD19 (**G**); on combined staining for kappa and EBER (**H**), there are many EBV+ cells that mostly correspond to the B cells, while most kappa+ plasma cells were EBER-negative (**H**); the LN also comprises prominent arborising vessels (**I**) and CD21 highlights expanded follicular dendritic cells (**J**); CD3 reveals a diffuse T-cells infiltrate (**K**) positive for several TFH markers (not shown)

Prominent B-cell expansions may even obscure the underlying TFHL. Three such cases were submitted (LYWS-1080, 1137, and 1453). Case LYWS-1137 presented by Nives Zimmermann was a 30-year-old man with lymphadenopathy, fevers, pleural effusion, and rashes. The patient underwent two LN core biopsies, leading to a diagnosis of an EBV+ lymphoproliferative disorder, which was initially treated with rituximab, but he progressed under treatment. In a subsequent surgically excised LN (Fig. 7A–C), the histopathological features were typical of AITL, including FDC expansion and a proliferation of atypical CD3+ CD4+ T cells with clear cytoplasm expressing CD10, BCL6, and PD1. A component of EBV-positive large PAX5+ CD30+ B cells was present but not prominent, likely attenuated by the treatment. This case was particularly unusual because it occurred in a young adult at an age where a diagnosis of TFHL is unlikely, which must have contributed to missing the diagnosis compounded by the initially limited sampling. Mutations were found in *RHOA* (G17V), *IDH2* (R172K), *TET2* (two mutations), and *STAT6* (variant of unknown significance), with variant allele frequencies in the range of 18 to 24%, suggesting that they were all somatic. Case LYWS-1080 submitted by Katrin Kurz (Fig. 7D–K), occurred in a more typical clinical setting in an 81-year-old woman with altered general condition, pleural effusions, generalized lymphadenopathy, and splenomegaly. The histological picture of the LN biopsy was dominated by a polymorphic infiltrate of large EBV+ B cells and monotypic EBV-kappa-positive plasmacytic cells. Clues to identification of the underlying AITL were the presence of arborizing high endothelial venules, expanded CD21+ FDCs and clusters of CD3+ CD4+ BCL6+ PD1+ ICOS+ atypical clear cells. PCR-based clonality studies showed an oligoclonal pattern for TR genes and monoclonal IG rearrangements. HTS found mutations typical of TFHL (*TET2*, *DNMT3A*, and *IDH2* R172K), and no additional variants that could be ascribed specifically to a B-cell component. The consensus diagnosis was AITL with associated monoclonal EBV-positive lymphoplasmacytic proliferation. This designation was preferred over considering two independent diagnoses, i.e., AITL and B-cell lymphoma, because the two components were intermixed, and because B-cell expansions are considered part of the biology of TFHL, with a clonal relationship to the T-cell neoplasm due to shared mutations originating in clonal hematopoiesis having been formally demonstrated in several cases [49, 52–54].

### Association of TFH lymphoma with clonal hematopoiesis (CH) and myeloid neoplasms

Up to 80% of patients with TFHL have underlying CH, with shared *TET2* and/or *DNMT3A* mutations in the early progenitor cells (i.e., hematopoietic stem cells) as well as the lymphoma [55–58]. CH appears to be the source of the increased incidence of myeloid neoplasms after TFHL-directed therapy [56, 58, 59]. Several studies have shown that while *TET2* and *DNMT3A* mutations are present in both myeloid and T-cell compartments, *RHOA* and *IDH2* mutations appear confined to the neoplastic T cells [58, 60, 61], suggesting that additional mutations are needed for lymphomagenesis.

**Fig. 8:**
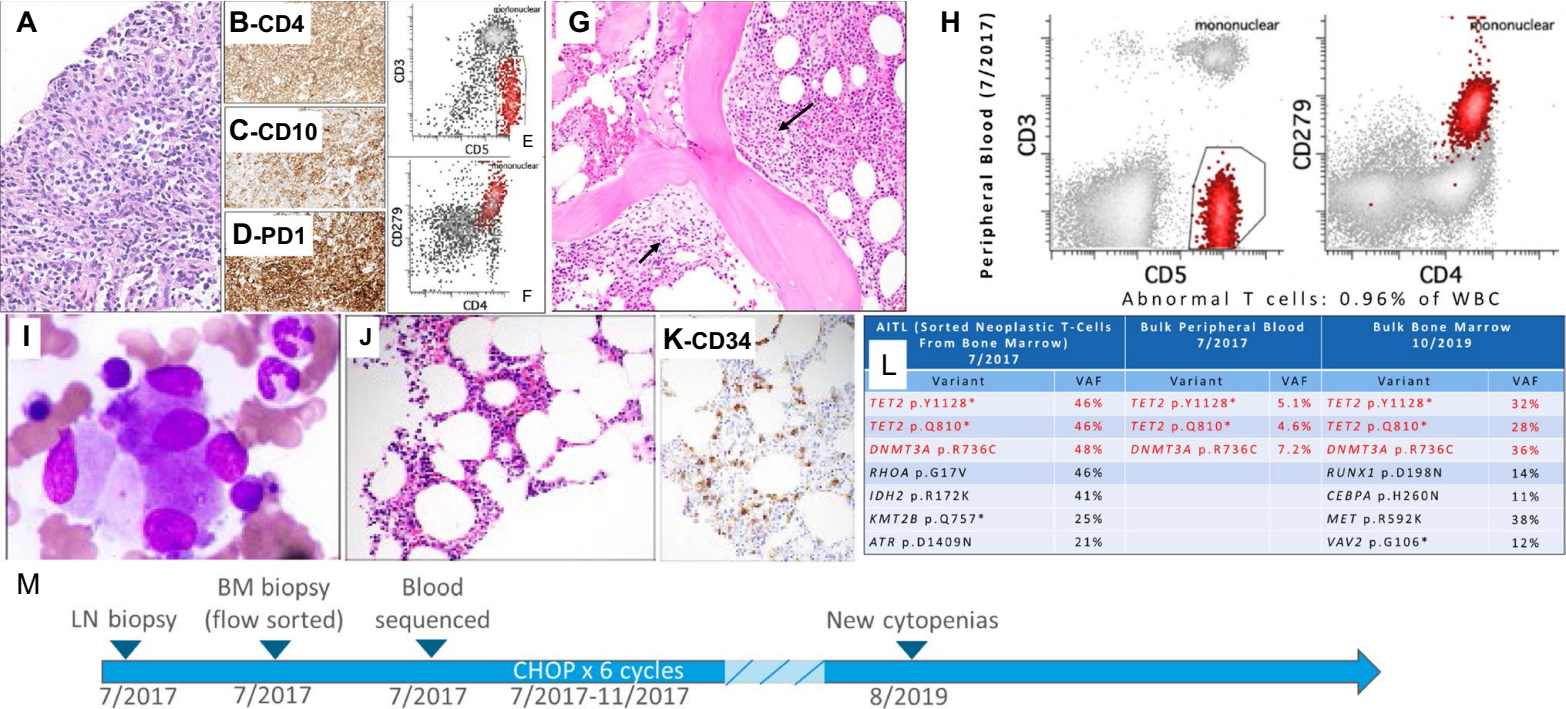
Divergent evolution of clonal hematopoiesis to angioimmunoblastic T-cell lymphoma and therapy-related myeloid neoplasm. LYWS-1247 (Natasha Lewis): 72-year-old male diagnosed with AITL on core needle biopsy (**A**), positive for CD4 (**B**), CD10 (**C**), and PD1 (**D**) by immunohistochemistry and with surface loss of CD3 by flow cytometry (**E**, **F**). A staging bone marrow biopsy demonstrated involvement (arrows, **G**) and bulk peripheral blood flow cytometry showed an exceedingly low level of involvement (**H**). Two years later, a bone marrow biopsy demonstrated dysmegakaryopoiesis (**I**), with hypocellular bone marrow (**J**) and increased blasts (CD34, **K**). Two *TET2* mutations and a *DNMT3A* were shared between the staging bone marrow involved by AITL, the bulk peripheral blood at diagnosis, and bulk bone marrow two years later, but only the specimen with morphologic AITL involvement demonstrated the *RHOA* p.G17V and *IDH2* p.R172K, and the bone marrow with myeloid progression acquired additional mutations (**L**). Timeline of events (**M**)

Five cases submitted to the workshop demonstrate this relationship between TFHL and CH or myeloid neoplasm. All patients with available HTS data demonstrated a shared *TET2* mutation between the myeloid and lymphoid compartments. The T-cell diagnosis was AITL in four cases, and a clonal T-cell lymphoproliferation suspicious for a TFHL in one case. Case LYWS-1389 (Erica Swenson) had a concurrent diagnosis of myelodysplastic syndrome (MDS) and AITL with a shared *TET2* mutation, and with *DNMT3A* and *RHOA* mutations additionally identified only in the AITL component involving the pleural fluid. Case LYWS-1156 (Yahya Al-Ghamdi) was a patient with clonal cytopenia of undetermined significance preceding a typical AITL. A small monoclonal B-cell population was also identified in the staging bone marrow biopsy, and a *TET2* mutation was shared between the LN and bone marrow. While the bone marrow cells were not sorted for genetic analysis, it raises the question of whether the *TET2* mutation was also shared by the B-cell clone, as described previously in AITL patients with *TET2* mutations identified in microdissected B-cells from tissue sections [62]. Presented orally was LYWS-1247 by Natasha Lewis (Fig. 8A–M), which elegantly demonstrated the relationship between CH, AITL, and myeloid neoplasms. The patient showed shared *TET2* and *DNMT3A* mutations in sorted neoplastic T-cells and bulk peripheral blood, with *RHOA* p.G17V and *IDH2* p.R172K mutations in the AITL cells. The bone marrow biopsy did not show morphologic evidence of a myeloid neoplasm at diagnosis. After chemotherapy for AITL, the patient developed new cytopenias, due to a therapy-related myeloid neoplasm (MDS with excess blasts—2, WHO4R-2017), with the same *TET2* and *DNMT3A* mutations previously identified, and with the acquisition of additional abnormalities in *RUNX1* p.D198N and *CEBPA* p.H260N.

## Other T-cell lymphoma entities or other lymphoproliferative disorders

### Peripheral T-cell lymphoma, NOS

PTCL, NOS is a mature T-cell lymphoma that does not meet the criteria for any other specific entity. GEP studies have identified two molecular subgroups within PTCL, NOS that resemble Th2 and Th1 cell differentiation profiles, known as PTCL-GATA3 and PTCL-TBX21, respectively. These subgroups have demonstrated differences in their morphological, molecular, and clinical characteristics [10, 63–65]. The lymphoepithelioid variant of PTCL, NOS (Lennert lymphoma) is characterized by the abundance of often clustered epithelioid histiocytes intermingled with neoplastic cells reportedly derived from CD8+ cytotoxic T cells [66, 67]. Over time, PTCLs with Lennert-like morphology were identified with overlapping features of AITL or with varying CD4+ or CD8+ immunophenotypes [68, 69]. Studies that have re-evaluated lymphoepithelioid PTCLs using a set of TFH markers revealed many, but not all, of them to be within the spectrum of TFHL [47, 70, 71], indicating that Lennert-like morphology can be seen in TFHL.

The boundary between PTCL, NOS and TFH, NOS is currently established based on the expression of a minimum two TFH markers. However, some series have reported that 20–30% of PTCL-NOS cases may express a single TFH marker [10, 72, 73] and the significance of this limited expression has yet to be fully investigated. Additionally, there are similar mutations in epigenetic regulators (such as *TET2* and *DNMT3A*) in cases of PTCL, NOS, particularly in the PTCL-TBX21 subgroup, which includes a subset with a cytotoxic phenotype [74, 75]. The availability of HTS studies and a comprehensive panel of TFH markers in the workshop cases can provide further insights into the boundaries between TFH-NOS and PTCL, NOS and enhance our understanding of the usefulness of mutational profiles in distinguishing between these entities [76].

**Fig. 9:**
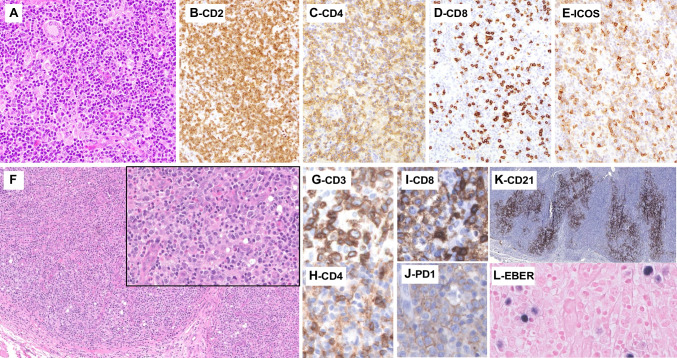
Peripheral T-cell lymphoma, NOS with overlapping TFH features. (Upper row) LYWS-1061 (Megan Fitzpatrick): 68-year-old male with three months of B symptoms, splenomegaly, and stage III lymphadenopathy. H&E shows diffuse small- to medium-sized abnormal lymphocytes and epithelioid histiocytes with occasional giant cells, imparting a Lennert-like appearance. (**A**) The lymphoma cells are positive for CD2 (**B**) and CD4 (**C**), negative for CD8 (**D**), and have partial expression of ICOS (**E**). Other TFH markers were negative. NGS identified two *TET2* variants and *NOTCH2* p.Gln2285Ter (c.6853C>T). There were insufficient TFH markers for a diagnosis of TFH lymphoma. (Lower row) LYWS-1401 (Anne-Roos Schrader): 44-year-old male with stage III lymphadenopathy, and splenic and tonsillar involvement by lymphoma. H&E shows a polymorphous infiltrate with intermediate-sized abnormal lymphocytes, eosinophils, and arborizing blood vessels, imparting an AITL-like appearance (**F**). Lymphoma cells are positive for CD3 (**G**). CD4 stains background smaller T-cells and histiocytes (**H**). CD8 is positive in the atypical lymphocytes (**I**), and there is partial PD1 coexpression (**J**). ICOS and BCL6 were also positive. CD21 demonstrated expanded and proliferated FDC meshworks (**K**). EBER-CISH showed scattered positivity (**L**)

Six cases submitted as “PTCL with expression of TFH markers or TFH immunophenotype,” were ultimately reclassified by the panel as PTCL, NOS. Three of these cases had lymphoepithelioid features. LYWS-1061 submitted by Megan Fitzpatrick (Fig. 9A–E) lacked sufficient TFH marker expression (only CD4 and ICOS) to diagnose TFH lymphoma. LYWS-1313 (Luis Colomo) was difficult but we could not unambiguously identify a neoplastic population convincingly positive for TFH markers (Supplemental Table 3). The third case with Lennert-like features (LYWS-1191, Siok-Bian Ng) was an unusual relapse of a PTCL, NOS with cytotoxic molecule expression at diagnosis and a background with many TFH cells. Moreover, none of these cases contained mutations characteristic of TFH lymphoma (Supplemental Table 2).

Presented orally by Lianne Koens, LYWS-1401 was an unusual PTCL with AITL-like histology, including clear cells with mixed inflammation, arborizing blood vessels, and prominently distorted follicular dendritic cell meshworks (Fig. 9F–L). The tumor cells were CD8+, and despite ICOS, BCL-6, and partial PD1 expression, this conflicted with the proposed COO of TFHL and, thus, is considered not diagnostic of AITL, Additionally, the constellation of mutations in this case (*STAT3*, *NOTCH2*, *VAV1*, *TBL1XR1*, and *CSF3R*) is not typical for TFHL (Supplemental Table 2) and a diagnosis of PTCL, NOS with features of AITL was favored. Interestingly, potentially activating mutations in the intracellular domain of *NOTCH2* were additionally found in two other PTCL, NOS described above, (LYWS-1313 LYWS-1061) both of which showed Th1-differentiation/PTCL-TBX21 by the immunohistochemical algorithm [65].

The last two cases in this subsection fulfilled the criteria for PTCL, NOS, despite each demonstrating positivity for two TFH markers. Case LYWS-1393 (Samah Kohla) demonstrated large cell cytology, loss of many T-cell antigens including CD4 and CD8, and no CD30 expression. The tumor was cytotoxic (perforin+) with coexpression of PD1 and BCL6. Case LYWS-1398 (Saumyaranjan Mallick) consisted of multiple large, extranodal tumoral masses weakly positive for CD4 with a partial TFH phenotype (PD1 and weak BCL6). The morphologic features and clinical presentation were inconsistent with a diagnosis of one of the recognized subtypes of TFHL. These two cases caution that the expression of two TFH markers should be in the context of a CD4+ phenotype and that additional more specific criteria may be required to establish a diagnosis of TFHL [36].

### Cutaneous T-cell lymphoma (CTCL)

**Fig. 10:**
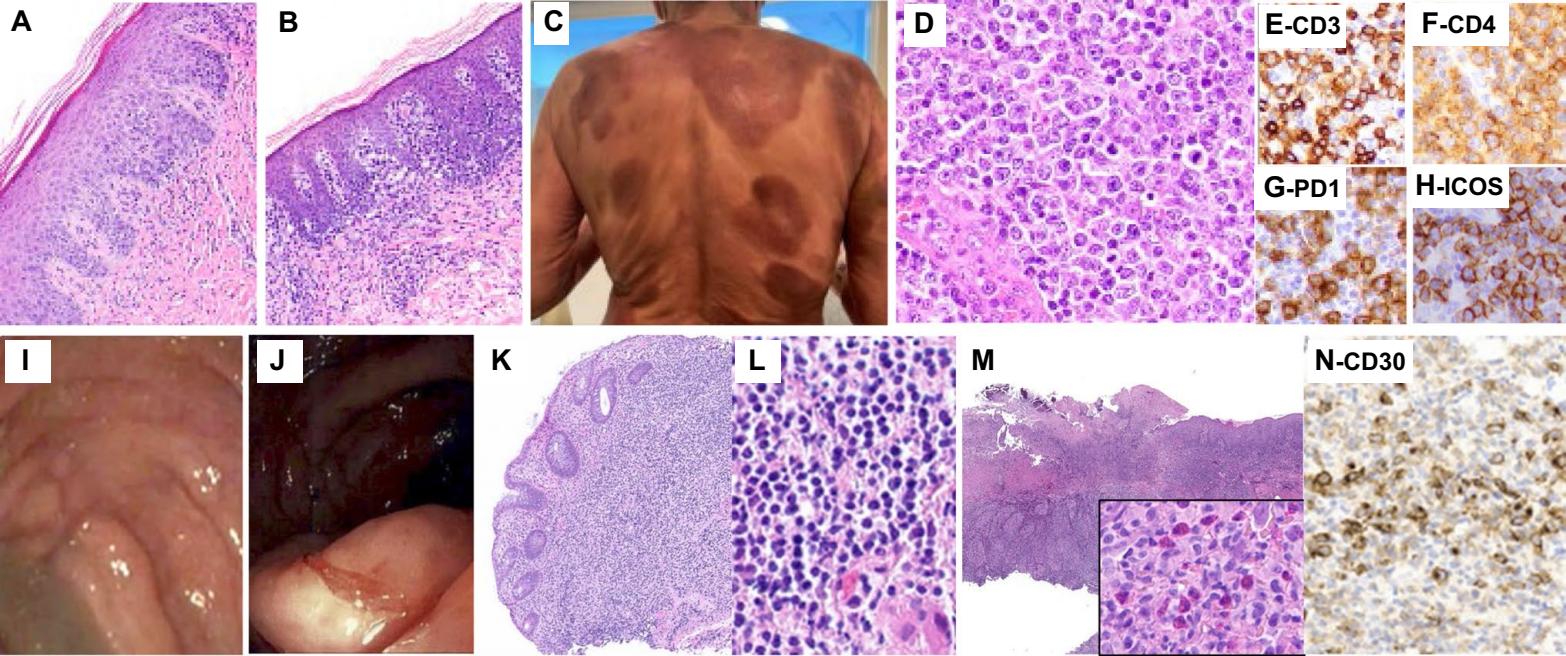
Cutaneous T-cell lymphoma and reactive lymphoproliferations can express TFH markers. (Upper row) LYWS-1174 (Yi Zhou): 78-year-old female diagnosed with cutaneous T-cell lymphoma 4 years ago (**A**) with worsening skin lesions (**B**, **C**), and generalized lymphadenopathy. LN biopsy demonstrated a diffuse proliferation of large neoplastic lymphocytes (**D**), positive for CD3 (**E**), CD4 (**F**), PD1 (**G**), ICOS (**H**), CD10 (subset), BCL6 (subset), and negative for CD7. A clonal TR gene rearrangement was identified. A *TP63*::*ARID5B* fusion was identified. No mutations in *RHOA*, *IDH2*, *DNMT3A*, or *TET2* were detected. (Lower row) LYWS-1197 (Philip Butlerys): intestinal TFH lymphoproliferation in a patient with activated phosphoinositide 3-kinase delta syndrome in a 14-year-old girl on IVIG with recurrent infections, chronic stable lymphadenopathy, and abdominal pain and diarrhea. Colonoscopy revealed areas of nodularity (**I**) and inflammation with ulceration (**J**). Histologically, multiple areas of the bowel showed a monotonous small lymphoid infiltrate (**K**, **L**) positive for CD3, PD1 (subset), and ICOS. LYWS-1212 (Carlo Pescia): a CD30-positive lymphoproliferation consistent with TUGSE in a 38-year-old HIV-negative male with sudden onset of oral aphthous ulcerations (**M**), composed of abnormally enlarged lymphocytes with associated eosinophils (inset). Many T-cells were positive for CD3, CD4, and PD1 and negative for cytotoxic markers and EBV. A clonal TR gene rearrangement was identified. CD30 (**N**) is positive in many of the lymphocytes, which has been reported in cases of TUGSE

CTCL with LN involvement is critical to distinguish from other subtypes of PTCL since management and treatment protocols differ substantially [77]. Mycosis fungoides (MF) and other primary CTCL frequently derive from the CD4+ T-helper subsets. A strong association with TFH immunophenotype has been previously demonstrated in the majority of cases of MF [78] and in primary cutaneous CD4+ small/medium lymphoproliferative disorder, in which positivity of up to 3 TFH markers was demonstrated in 92% of cases [79]. That said, AITL can involve the skin, and expression of TFH markers and detection of *RHOA* p.G17V and *IDH2* p.R172K mutations in this context can suggest the differential diagnosis if staging information is not yet available [80]. Three cases submitted to the workshop as nodal TFHL were re-classified as primary CTCL with secondary LN involvement. The LNs LYWS-1174 (Yi Zhou) and LYWS-1349 (Gorana Gasljevic) were each preceded by a history of MF. The LN morphology showed non-specific effacement by intermediate to large-sized T cells with clear cytoplasm (LYWS-1174) or effacement by dermatopathic areas (LYWS-1349). CD4+ neoplastic cells in both cases expressed three TFH markers (PD1+ and ICOS+, plus BCL6 or CXCL13). Interestingly, case LYWS-1174 harbored a *TP63* rearrangement, which has been previously described in other PTCLs including ALK-negative anaplastic large-cell lymphoma, PTCL, NOS, and transformed MF (Fig. 10A–H) [81, 82]. The panel considered this case to represent secondary nodal involvement by transformed MF. Case LYWS-1349 demonstrated composite mantle cell lymphoma, together with nodal involvement by CTCL. LYWS-1263 (Bei Yang) submitted as TFHL involved a 74-year-old female with fatigue, weight loss, splenomegaly, and extensive lymphadenopathy. She also had a diffuse rash, WBC count of 22.7 × 10^9^/L, negative HTLV1 serology, and rapidly progressive, fatal disease. The LN demonstrated interfollicular lymphoid infiltrates of hyperchromatic cells with convoluted nuclei and nucleoli, positive for CD4, PD1, BCL-6, ICOS, and TP53. HTS found mutations in *TP53* c.733G>A (22% VAF) and *CCR4* c.971dup (17% VAF). While it is difficult to know with certainty, the pattern of infiltration, probable leukemic involvement, and mutational profile are most consistent with a CTCL with leukemic presentation, such as Sézary syndrome.

### Adult T-cell leukemia/lymphoma (ATLL)

ATLL has a myriad of clinical presentations and can mimic several subtypes of PTCL, including harboring TFH immunophenotype or AITL-like features [83]. To avoid misdiagnosis, it is crucial to perform systematic HTLV1 serology, particularly in areas where the HTLV1 virus is endemic. Adrien Pecriaux presented case LYWS-1359, which involved a 62-year-old man of Caribbean ancestry with high-stage lymphadenopathy and splenomegaly. An inguinal LN biopsy appeared as a tumor-cell-rich AITL, with abundant lymphoma cells, clear cytoplasm, and arborizing vessels, and expressed a TFH immunophenotype (CD3+, CD5+, CD7-, CD4+, strong ICOS, and PD1), though CD25 and FOXP3 were also positive. The FDC meshworks were not expanded, and EBER-CISH was negative. Positive HTLV-1 serology was observed, and HTS characterization of HTLV-1 integration sites disclosed a major clone identical in the LN and blood [84]. Interestingly, an *IDH2* R172K variant was identified, questioning whether this could be responsible for the clear cell cytomorphology. However, other characteristic mutations typical of TFHL were not detected.

### Others

Other types of lymphoma and lymphoproliferative disorders can mimic TFHL or demonstrate a partial TFH immunophenotype [72]. Case LYWS-1394 (Valentina Tabanelli) was a classic example of a lymphoid variant of hypereosinophilic syndrome (L-HES) in a 51-year-old man with a unilaterally enlarged tonsil and a 13-year history of eosinophilia and an aberrant CD4(+), sCD3(-) T-cell population in the peripheral blood. Aside from isolated tonsil enlargement, the patient was asymptomatic and whole-body PET scan was negative. Identical T-cell immunophenotypes and matching clonal amplicons were identified in the blood, bone marrow, and tonsil, demonstrating near-total effacement by abnormal T-cells expressing partial/weak PD1 and ICOS. CD10, BCL6, and CXCL13 were negative. TFH marker expression is not demonstrated in cases of L-HES and may indicate an evolving sCD3-/CD4+/TFH+ AITL in the differential diagnosis [85, 86]. Despite weak/partial TFH expression in this case, the localized presentation in one tonsil with no microenvironment of a T-cell lymphoma contributed to the panel diagnosis of L-HES rather than TFHL. Finally, abundant reactive TFH cells can occur in various B-cell lymphomas and lymphoproliferative disorders [72, 87]. Examples were case LYWS-1233 (Tanuja Shet) with a variant pattern of nodular lymphocyte predominant B-cell/Hodgkin lymphoma, and case LYWS-1463 (Tiago Maia) with a relapsed EBV-associated polymorphic nodal lymphoproliferative disorder [72, 87].

## Reactive or other lymphoproliferations

Several cases were favored to represent reactive lymphoid proliferations of TFH cells (Supplemental Table 4). Case LYWS-1197 (Philip Bulterys, Fig. 10I–L) was a colonic biopsy from a 14-year-old girl known to have an inborn error of immunity due to a mutation in the *PIK3CD* gene giving rise to activated phosphoinositide 3-kinase Δ syndrome (APDS-1). She presented with chronic stable lymphadenopathy, abdominal pain, and diarrhea. Biopsies showed prominent diffuse lymphoid hyperplasia comprising monotonous small T-lymphocytes expressing CD4, PD1, CXCL13, and ICOS in multiple foci of bowel. Lymphoproliferations of TFH cells in the context of APDS-1 have been previously described [88] including a case report with a similar intestinal nodular lymphoid hyperplasia that resolved after treatment with sirolimus [89]. Carlo Pescia contributed LYWS-1212 involving the sudden onset of oral and lingual aphthous ulceration in a 38-year-old male composed of CD3+ CD4+ PD1+ and frequent CD30+ lymphocytes and eosinophils with a monoclonal TR gene rearrangement that spontaneously regressed; the panel considered this as a form of CD30+ lymphoproliferative disorder most consistent with traumatic ulcerative granuloma with stromal eosinophilia [90] (Fig. 10M and N). This case had only one positive TFH marker, stressing the importance of using multiple TFH markers when assigning TFH lineage and considering the clinical context when entertaining a diagnosis of TFHL. Finally, two submitted cases represented non-specific reactive lymphoid hyperplasia in LN (LYWS-1145 by Tapan Bhavsar and LYWS-1254 by Wen-Qing Yao) with increased numbers of TFH cells as part of a paracortical polymorphic infiltrate or florid follicular hyperplasia.

## Conclusions

This spectrum of cases submitted embraced the difficulty in diagnosing TFHL and highlighted notable characteristics of TFH subtypes, as well as reactive and neoplastic mimics (Box 1). The cases reflect the broad differential diagnosis of TFHL and emphasizes the importance of clinical correlation. The final distribution of session V workshop cases had 25 out of 42 cases (60%) being confirmed to as belonging to one of the three follicular helper T-cell lymphoma subtypes (16 AITL-type, 3 TFHL-F, and 6 TFHL-NOS), reflecting the difficulty in assessing the TFH immunophenotype, its lack of specificity, and the need for caution.

**Table 1:** **Box 1**

• A TFH immunophenotype is defined by the expression of a minimum of two TFH markers (of the five recommended for testing, i.e., CD10, BCL6, PD1, ICOS, and CXCL13) in CD4+ T cells • The lower end of the clinicopathologic spectrum of TFHL includes cases with low tumor burden/early involvement and/or pauci-symptomatic, slowly progressive, or indolent disease • Diagnostic difficulties in TFHL include the presence of a B-cell component mimicking cHL, cutaneous T-cell lymphoma expressing TFH markers, and reactive TFH proliferations as part of other lymphoproliferative disorders or benign hyperplasias • TFHL and myeloid neoplasms share an association with underlying CH • Separating TFHL and PTCL, NOS requires understanding the morphologic spectrum of TFHL, demonstrating a strong CD4+/TFH+ immunophenotype, and the presence of certain mutations that are recurrent in TFHL • Needle core biopsies pose a risk to accurate and timely diagnosis of TFHL • Supportive tests including flow cytometry and PCR or genomic testing are helpful in challenging cases especially those with low tumor burden; these contributed to the final diagnosis of 25 cases in this series	

### Supplementary information

ESM 1 (XLSX 100 kb)

## Data Availability

This is not applicable.
